# Making the post-MDG global health goals relevant for highly inequitable societies: findings from a consultation with marginalized populations in Guatemala

**DOI:** 10.1186/1475-9276-13-57

**Published:** 2014-10-10

**Authors:** Ana Lorena Ruano, Silvia Sánchez, Fernando José Jerez, Walter Flores

**Affiliations:** Center for International Health, University of Bergen, Bergen, Norway; Centro de Estudios para la Gobernanza en los Sistemas de Salud, Guatemala City, Guatemala

**Keywords:** Guatemala, Community participation, Community consultations, Go4Health, Millennium development goals, Sustainable development goals

## Abstract

**Introduction:**

The United Nations presented a set of Millennium Development Goals that aimed to improve social and economic development and eradicate poverty by 2015. Most low and middle-income countries will not meet these goals and today there is a need to set new development agenda, especially when it comes to health. The paper presents the findings from a community consultation process carried out within the Goals and Governance for Global Health (GO4Health) research consortium in Guatemala, which aims to identify community needs and expectations around public policies and health services.

**Methods:**

Through a participative and open consultation process with experts, civil society organizations and members of the research team, the municipalities of Tectitan and Santa Maria Nebaj were selected. A community consultation process was undertaken with community members and community leaders. Group discussions and in-depth interviews were conducted and later analyzed using thematic analysis, a qualitative method that can be used to analyze data in a way that allows for the identification of recurrent patterns that can be grouped into categories and themes, was used.

**Findings:**

Following the Go4Health framework’s domains for understanding health-related needs, the five themes identified were health, social determinants of health, essential health needs and their provision, roles and responsibilities of relevant stakeholders and community participation in decision-making. Participants reported high levels of discrimination related to ethnicity, to being poor and to living in rural areas. Ethnicity played a major role in how community members feel they are cared for in the health system.

**Conclusion:**

Achieving health goals in a context of deep-rooted inequality and marginalization requires going beyond the simple expansion of health services and working with developing trusting relationships between health service providers and community members. Involving community members in decision-making processes that shape policies will contribute to a larger process of community empowerment and democratization. Still, findings from the region show that tackling these issues may prove complicated and require going beyond the health system, as this lack of trust and discrimination has permeated to all public policies that deal with indigenous and rural populations.

## Introduction

In the year 2000, the United Nations (UN) presented eight Millennium Development Goals (MDGs) that aimed to improve social and economic development and to eradicate poverty. The three health-related goals sought to reduce child mortality, improve maternal health and fight against the HIV/AIDS epidemic, malaria and other diseases. These goals should be met by the year 2015. However, evidence suggests that most low and middle income countries will not be able to do this, and that countries that started off with worse indicators are still the ones that are lagging behind the most [1, 2]. The global consensus now is to renew the MDGs through the embracement of different goals that have clear accountability mechanisms built into them.

In 2011, the research consortium Goal and Governance for Global Health (Go4Health) was established with the mandate to provide evidence-based advice on the new health-related [3]. Because the MDGs were originally set as narrow and sector specific targets, they were not derived from an inclusive analysis or from the prioritized needs of the populations that suffered the highest burden of disease, poverty and social exclusion [2]. In addition to this, there were no accountability mechanisms or adequate participation strategies built into them [4]. As a way to deal with this, the consortium proposed to advance on the global social contract first presented in the Millennium Declaration through the articulation of new goals and by developing a governance structure with a clear framework of shared, but differentiated responsibilities [3, 5]. Part of this process includes engaging marginalized communities in meaningful participatory processes that identify their needs and priorities so that their voices can be a part of the setting of the new goals. In addition, through engaging these communities we also aim to contribute to the building of accountability mechanisms for the implementation and evaluation of these new goals.

Marginalization is a complex and active social process where historical, social, cultural and economic factors interact with the implementation of exclusionary policies in order to push a specific population towards the edge of society. The goal of this process is to isolate individuals, communities or populations from services, life opportunities and decision-making processes [6]. As a result, marginalized communities are less able to promote their interests and to be included in policy-making spaces, which in health translates into deficient health services, a lack of adequate human resources for health and weaker health systems. At the global health level, this has been translated into global goals that do not necessarily reflect the needs, priorities and goals of large sectors of the world’s population [4].

Today, there is a need for planning and developing a global work agenda that will allow for the improvement of the quality and access to health services for the world’s population. These new global goals should be based on the values, needs and priorities identified by many different communities so that their creation and completion can benefit a wider array of peoples at the global and the community level [3]. However, this can only happen through the development of a meaningful participation process that is able to gather these views as well as have comprehensive accountability mechanisms that will allow communities, civil society and other institutions to be included [7]. As part of the Go4Health community consultation process, this paper presents the findings from two community consultations carried out in the rural highlands of Guatemala. Our aim was to implement a process of consultation and engagement with marginalized communities in order to identify their needs and the expectations they may have about public polices and health services.

## Methods

### Setting

Latin America is one of the most unequal regions in the world in terms of income and wealth distribution, and it presents high levels of social exclusion and marginalization that is maintained through very low levels of social mobility. The region has ten out the 15 most unequal countries in the world and the UNDP has pointed out the importance of having pro-equity policies that include the voices of different population groups as a way to change this trend [8]. Inequality and marginalization affect women, indigenous groups and African descent populations harder than other groups. They earn less money, have less access to education and carry a higher burden of disease than other groups [9].

Guatemala is an ethnically diverse, Central America country with 14 million in habitants, of whom 52% live in poverty and 75% live in rural areas [10–14]. It is a middle income country with a Gini coefficient of 53.7, and the World Bank named it one of the most unequal countries in the world [13, 14]. Indigenous people make-up 45% of the population, and they live in a disadvantageous position in comparison to their non-indigenous counterparts: it is indigenous groups that tend to live in rural, geographically isolated places where there is little access to health and other social services [15–17]. This isolation also restricts their capacity to participate in policy-making processes.

#### Tectitan

Located in the province of Huehuetenango, this municipality has 8,025 inhabitants of whom 28.5% are indigenous. Most of the population lives in rural areas, 70% of them are poor and 28% are extremely poor [11, 12]. Subsistence farming is the main economic activity and only about 10% of the people in Tectitan can cover the basic household expenses. Remittances from relatives in the United States are another major form of income, with 25% of the population receiving money from abroad [13, 18]. The widespread poverty is reflected in children, and 27% of them present severe stunting. When it comes to health services, there is a health center that is open around the clock, four health posts and fifteen community clinics that receive ambulatory physicians and nurses once a month [18].

#### Santa Maria Nebaj

The municipality of Santa Maria Nebaj, located in the province of Quiche, has a total of 73,218 inhabitants of whom 95% are indigenous and 35% live in rural areas. Of the total population, about 68.5% are poor and 29.5% live in conditions of extreme poverty [11, 12]. The main economic activity is subsistence farming and there are few opportunities for the production of marketable goods [13]. About 36% of the households lack access to at least one basic service, and most of the population does not have access to secondary or higher education. In addition, the lack of adequate roads and transportation mean that moving around is complicated, expensive and time consuming [19].

The district hospital, twelve health centers and 51 community clinics are not enough to provide health services for the population of Nebaj. In 2010, only 37% of the people in this town had access to the health system, with easier access for families living in urban areas. As a result, only 13% of births are carried out in an institutional setting. The rest of the births happen at home with Traditional Birth Attendants and with limited access to obstetric care [19].

### Data collection and analysis

The first step in this process was the selection of the municipalities whose population would be consulted. This was done through a participatory process where experts, civil society organizations and the members of the research team discussed and proposed different options regarding possible communities. Taking into account existing indicators on social exclusion and the work previously carried out by the research team, it was decided to consult community leaders and community members in the municipalities of Tectitan and in Santa Maria Nebaj.

After the communities had been selected and contacted, ALR, WF, SS and FJ developed a discussion guide based on the domains previously identified by the Go4Health research consortium. The data for this paper was collected over a week of intense fieldwork with community members and community leaders from the municipalities of Tectitan and Nebaj. ALR, SS and FJ collected the data. When possible, the interviews and discussions were conducted in Spanish. However, an interpreter with training in social sciences and in qualitative research methodologies led the group discussion with community members in Nebaj, and was also in charge of transcribing and translating this discussion. In total, there were five group discussions: three of them were conducted in Spanish with community leaders in Tectitan. Each of these had from eleven to fifteen participants, who were all community leaders. All the groups were made up of both women and men. In Nebaj, there were two group discussions. The group discussion with community members was carried out in the local language and had six participants. The group discussion with community leaders was carried out in Spanish and consisted of seven leaders. As in Tectitan, both groups consisted of men and women. The discussions were tape recorded and later transcribed. In the case of the group discussion in the local language, the translation was done before transcribing. All the participants were asked for their verbal informed consent, which was perceived as a more viable option given the context. In addition, consent was sought in order to make voice and video recordings, as well as to take photographic documentation of the data collection process.

Thematic analysis, a qualitative method that can be used to analyze data in a way that allows for the identification of recurrent patterns that can be grouped into categories and themes, was used [20, 21]. To do this, data was organized systematically through the identification of topics that link together the meaning units, or codes, into categories and later into mutually exclusive themes [21]. The first step was for ALR and WF to carefully read the transcripts. Afterwards, the data was coded. The emerging codes were then organized into a mix of emerging and a priori categories and later these categories were organized into the five themes selected by the Go4Health framework. A table of the original domains and categories used by Go4Health and for this study is presented below. The table only presents the domains and categories that originated from the Go4Heath framework as well as emerging ones. Finally, ALR and WF selected quotes that exemplify the main findings (Table 1).

**Table 1:** **List of Go4Health domains and categories**

***Domain***	***Categories***
Health	Community health
	Family health
	Personal health
	Being healthy
	Being sick
Social determinants of health	Holistic health
	Social determinants of health
	Alternative health
	Discrimination
Essential health needs and their provision	Basic and essential health care services
	Mechanisms to ensure access and quality
	Right to basic and essential health services
	Problems in accessing care
	Priority setting
	MoH and the Health system
	Private versus public care
	Patient abuse
Roles and responsibilities of relevant stakeholders	Stakeholder responsibility
	Stakeholder accountability
	Attitudes of health providers
	Care providers
Community participation in decision-making	Community monitoring
	Monitoring outcomes
	Community participation
	Leadership
	Incidence or impact of community participation

### Ethical considerations

The engagement and work with marginalized communities should be carried out within an ethical framework that reflects the precarious situation in which marginalized people live their day-to-day [22]. Their vulnerable position can limit the capacity to practice their autonomy [23, 24]. Informed consent was secured through informative sessions where our objectives and methodology were clearly discussed with all the participants, who were assured that their participation in this project did not endanger the benefits they are obtaining from the on-going partnership and work between their communities and our institution (CEGSS). The already existing relationship with the communities that participated in this study helped us to provide them with support through both the findings presented here and through other capacity building process currently being implemented that would help to ensure more inclusion in municipal-level decision-making processes. The findings as well as photographic documentation of the consultation and the discussions were shared with the community leaders so they could be used as evidence for community monitoring reports presented regularly at the municipal-level health commission in their municipalities.

## Findings

Here we present the findings from the consultation process carried out at Nebaj and Tectitan. The data is presented according to the Go4Health established domains, where each domain represents a theme in the analysis.

### Theme 1: health

Health is seen as ‘the good of the people’, and community health is a shared value that comes from the feeling of belonging to a community. The importance of community life, of sharing with neighbors and of teaching the value of a tight-knit community in school exemplifies this. The community is a place where you can trust your neighbors. Hygiene was directly related to health and being clean was the perceived solution to many health problems. Everyone in the community can contribute to the collective hygiene, since this is not related to the expenses or the use of resources but to personal practices. Environmental health was a central component of healthy communities. Having access to sanitation services and latrines as well as keeping water sources clear from pollution were all important.

Having a balanced life that included work, family, play and relaxation time as well as personal time to reflect were all seen as components to achieving personal health. Being healthy was defined as having good mental, physical and moral wellbeing. The home is seen as the central component to a healthy family. This is where health begins because it is the family that teaches good eating and hygiene habits. The family is also the one that should be in charge of caring for children and the elderly. The role of the parents in a family is to provide food, water and health for the younger and older generation. Care should be present in a way that is easily accessible for families, as community leader from Tectitan stated:

*‘… what we need in our community is medicine for our children. Kids are not fed well and then they get sick: diarrhea or nausea and maybe it goes on for more than twelve hours and they get worse. We don’t have medicine in our communities and doctors don’t treat us and send us to other places and then we lose our child’.*

In Nebaj, community members reported feeling that illnesses where more prevalent among the indigenous, Maya population. Although there were complaints about racism in health services in both municipalities, it was only in Nebaj where participants reported that the reason they are sick so often is their ethnicity, and that this is why they are so mistreated at the health facilities. As one community member from Nebaj shared:

*‘A pregnant Quiche woman went to the health center to deliver… but the nurse would not help her. Her husband had to all the work and no one paid attention to her. She delivered the baby on her own. This is because they don’t care about us, because indigenous people, we get sick a lot… They get tired that we are sick more often than non-indigenous people and they wish we all died’.*

### Theme 2: social determinants of health

Health is obtained from equality, development, from having respect for neighbors and culture, and from being able to lead a good life. However, this is hard to achieve because of the many factors that influence health that are beyond the community member’s control. This translates into feelings of helplessness. Water was seen as the main determinant for health because of its role in food preparation, as a drink and because without it, it is impossible to have a clean house and community.

Discrimination was widely reported by all the participants and encountering racist healthcare providers that did not speak the local language and treated indigenous patients badly was a common occurrence. This was a barrier to health and was attributed to community members being poor, rural and indigenous. Constant insults reported included calling patients dirty and family members being blamed for the health issues of their relatives. A community member from Nebaj:

*‘A person already knows how they will be treated* [at the health center]*, so the people in the villages always say that they don’t want to go to the hospital because they will argue with me. They’ll ask me why I look like I do, why I only came now that I’m so ill and why didn’t I come when I first got sick. They don’t think that the distance to the hospital is great for us. They don’t know how the road is or how much money we spent getting here. We have to think about going to the hospital…’*

In Nebaj, the high levels of discrimination that were reported mean that many of the study participants use alternative medicine, be it natural medicine or through treatment from traditional healers. In many of the cases from this municipality, traditional medicine was perceived as more effective than western medicine. This is because herbs seem to be more readily available than drugs. Community members also reported that getting an injection or being treated at the community usually worked better than western drugs, and prayer was also highly valued as a tool against being sick.

### Theme 3: essential health needs and their provision

The participants of this study state that the biggest barrier to receiving care was the lack of drugs in both health services and pharmacies. When drugs are available, the lack of brand names was an issue. This is because generic drugs are seen as not as powerful as their brand-name counterparts. This lack of supplies, long waiting times and the lack of care for babies and children, as well as the lack of a free ambulance for patient transport were all issues commonly encountered at basic health facilities and hospitals alike. Furthermore, when drugs are available, they are only given to people that have personal connections to the healthcare providers and only seldom given because of need. A leader from Tectitan:

*‘…. A family was sick and they went for a consult. They got a prescription for the medicine and the doctor told the father ‘we don’t have any drugs’, and so the man left and when he was leaving he ran into his friend that works in the health center. The friend told him to wait and he came back with boxes of medicine for his friend. He said ‘look, there’s medicine in there and there’s more but I can only give you this’. The father of the family accepted because the medicine was really expensive… that’s how the health center is. They sell the medicine to the pharmacies and don’t give them to the people’.*

The need for health facilities that could provide quality services with culturally friendly staff was important to the participants of this study. Having doctors, nurses and hospitals that would provide good care would help to balance out the long waiting times. However, the services now are deficient and patients report being routinely turned away and denied care. This happened either because only ‘sick looking patients’ are cared for or because patients fall outside of the priority, which is maternal health and children under five.

The distance to the health facility is a major barrier to receiving healthcare and the MoH doesn’t always provide transport for free. There are very few working ambulances or vehicles for mobilizing patients and these are usually unfit to go into the most geographically secluded areas. As a result, it is only families that live in urban or peri-urban area that can use this resource. Rural families have to rely on private transportation and many get overcharged for this service or are told the price increased halfway through the trip. Once the patient arrives at a health facility, it is common to experience verbal abuse from healthcare providers. Patients reported being threatened and blackmailed. They were also charged hidden fees for drugs or care and reported being neglected.

Community members in Nebaj and Tectitan see health as a right, and report that keeping track of complaints is a good way to ensure access and quality of care. This is not an easy task because people from the communities have little power when compared to the health staff. However, demanding rights, modern facilities and good services is a community priority.

The health system, and specifically the MoH, is perceived as helpless because of its lack of resources. Community members report that institutions do not do their jobs correctly. There is a lack of trust in the health system and many community leaders supported the idea of involving the municipal government in the work that the MoH does locally. This would improve the way priorities are selected because according to community leaders in both municipalities, the health priorities now do not reflect their needs and are not chosen in a consultation process with them. In Tectitan, care is so deficient that many community members routinely cross the border over to Mexico to receive care there.

### Theme 4: roles and responsibilities of relevant stakeholders

In general, health care providers are perceived as incompetent: doctors and nurses, whether they are indigenous or not, routinely avoid their work responsibilities and ignore patients. Community leaders and community members from both municipalities reported cases of corruption, where nurses and doctors had been caught selling MoH drugs that should be available for free. As a result, these healthcare providers are perceived to be selfish and like they do not care for the community they are there to serve. The reported over-use of interns contributes to this negative perception of doctors, and according to community members, the positions in the health centers are not filled with the most capable and responsible person but by the one that had the most clout. Community members argued that because their salaries are paid through taxes, doctors and nurses are accountable to them, even if the healthcare providers do not recognize this.

The lack of adequate care and compassion towards patients has made doctors and nurses be perceived as only caring about their paycheck and not about their work. Doctors and nurses are routinely rude and racist towards patients and families and provide deficient care. Health authorities are perceived as arrogant and they do not share budgets or policy-related information with the community. In Nebaj this behavior, along with the corruption charges over MoH drugs and the racist attitude of the healthcare providers has led communities to think that doctors and nurses treat so badly because they want indigenous patients to die. A community member from Nebaj:

*‘… a really sad case because the woman is no longer with us. She was 16 and she was having her first baby* [in the hospital] *and the TBA didn’t go with her. The girl didn’t have any experience, her mother did but only with having her children, not with helping women have babies. They* [the health staff] *left her in a gurney, where she had the baby and died alone. Ten minutes later the baby died. They both died and no one cared… I don’t think it was disease that killed that poor woman. Her husband is now alone and heartbroken. Was it a lack of equipment? No, I don’t think so…’*

The MoH is still seen as the leader in health, despite its shortcomings. Community leaders and members agree that it is up to the ministry to lead the way for all the stakeholders in the health sector. Communities have the duty to be organized into development councils and health commissions and their role is to monitor health authorities and facilities. Doctors and nurses should be accountable for their work and should regularly inform the communities they serve about budgets, targets and other important information. However, the intense resource constraints that the health facilities face, the lack of accompaniment from office of the human rights ombudsman, the fact that most doctors and nurses work for several months with no paycheck and the high rotation of community leaders all make it hard to implement actions or change.

### Theme 5: community participation in decision-making

Community participation was highly valued in both Tectitan and Nebaj because it is only through it that the whole community can change and improvement can come. Participation solves problems and connects community members, and it is thanks to it that health and rights can be achieved and safeguarded. By participating together, community members and leaders reported a tightening of community bonds. However, not everyone participates and in order for this to become a widespread practice more support from institutions and from the government is needed.

There are many perceived benefits to participation: it leads to development and change, and this improves the community and the country. This is because community members’ rights are only respected when they speak out, so making demands on authorities and institutions is good. While the ability to make demands is positively perceived, community members stated that change does not happen on its own. Because authorities do not listen to their demands, the perception that participation does not bring about change is present among some community members. Many community leaders feel the same way because although they seek help from authorities and institutions, they seldom receive it. This is because they lack the power resources that would allow them to be a part of the decision-making. Community leader from Tectitan:

*‘… I want to ask you: what are we supposed to do? What can we do as leaders in our communities? Do we call everyone in our communities to tell them what we’ve talked about and how this is going to be a reality? On the contrary, if we tell our neighbors all the things we hear in the meetings they will get overwhelmed because they will think that nothing can be done. The mayor is the one that has the power, he is the one that decides what can be done and what can’t be done.’*

Community monitoring is viewed as an important activity that is carried out by community organizations such as the health commission or the development council. The leaders agree that their role should be to monitor and help community members file complaints about deficient care or ill treatment. However, people are afraid to file complaints and report lacking the tools to gather evidence. Without proper tools, community leaders argue, it is hard for them to negotiate with health and municipal authorities.

## Discussion

In Guatemala, many community members define health as a holistic concept that includes positive relationships with the environment, neighbors, friends and family [25, 26]. However, the country’s health system values the biomedical paradigm over community knowledge, and the long-standing power inequalities that affect indigenous and rural populations translate into the exclusion of non-conforming views. The marginalization of these peoples, both at the health system level and in other areas of society, has translated into very poor access to low quality care that does not reflect their worldviews or the values of the culture to which they belong [27]. Inclusionary practices that take into consideration values that go beyond those related to cost-effectiveness are a key part of the process of shaping a health system that more accurately reflects the values and needs of the population it is there to serve [28–30].

The discrimination that indigenous and rural community members reported experiencing when seeking health services is systemic in nature, and reflects long-standing racial issues in the country [16]. In addition, instances of abuse and neglect experienced by friends and neighbors further shapes the perceptions of individuals. This generates feelings of distrust towards the public health system that are based on ethnicity [31, 32]. In contrast, community members perceive that the private health system offers-up a more positive experience where the ability to pay for services is related to the quality of care received.

In addition to the experiences of discrimination, the participants’ experiences with health services that lack essential medical supplies, drugs and an emergency transport system has contributed to the breakdown of the relationship between the health system and the community. Studies have found that health systems in Latin America tend to cater to urban, and more affluent populations over rural, indigenous and poor ones [33]. However, a strong health system requires building on values such as fairness, trust and legitimacy, something that cannot be obtained through policies that are not perceived as impartial [30–32]. It is important to establish mechanisms of dialogue, consultation and participation, as it is through these practices that issues can be resolved and a more meaningful cooperation between stakeholders can occur.

Communities have tried to deal with the discrimination and lack of services by presenting complaints to local health and municipal authorities. However, the lack of response has led to the development of feelings of despair and hopelessness with community participation processes in some of the consulted people. The country’s progressive legal framework on participation offers the opportunity for communities to be involved in policy-making at the planning, implementation and monitoring stages. However, the vague language used and the lack of specific guidelines translate into participation processes that reflect local power dynamics and interests and not a straight-forward process that is similar all across the country [7]. The challenge is how to implement tools and procedures that can enforce and clarify this framework in a way that benefits marginalized and excluded populations, as well as how to implement clear accountability mechanisms that could provide much needed support for the communities and citizens that engage with the health system.

Despite the negative feelings reported by community members in both municipalities, their continued engagement in municipal and community-level health commissions shows that there is a willingness to continue to demand the fulfillment of the right to health. Organized communities that are able to use the existing legal framework and participation structure can be a part of decision-making processes from which they would have been excluded otherwise [34].

## Conclusion

Achieving health goals in a context of deep-rooted inequality and marginalization requires going beyond the simple expansion of health services and working with developing trusting relationships between health service providers and community members. In order to do this, long-standing ethnic, social and economic inequities need to be addressed. In addition, involving community members in decision-making processes that shape policies will contribute to a larger process of community empowerment and democratization. Still, findings from the region show that tackling these issues may prove complicated and require going beyond the health system, as this lack of trust and discrimination has permeated to all public policies that deal with indigenous and rural populations.
